# Effects of exercise intervention on physical mobility in stroke patients: a scoping review and research progress

**DOI:** 10.3389/fneur.2025.1609242

**Published:** 2025-08-05

**Authors:** Shiguang Ren, Qiliang Wan, Yijing Liu

**Affiliations:** ^1^Department of Sport Science, College of Natural Sciences, Jeonbuk National University, Jeonju, Republic of Korea; ^2^School of Martial Arts, Henan University, Kaifeng, Henan Province, China

**Keywords:** exercise intervention, stroke, physical mobility, neuroplasticity, muscle strengthening

## Abstract

**Aim:**

The aim of this study is to review the effects of exercise intervention on physical mobility in stroke patients and to explore its potential mechanisms in physical mobility in stroke. A scoping review of the literature was used to analyze the effects of relevant exercise interventions on the physical mobility of stroke patients.

**Results:**

Different exercise interventions significantly improves the physical mobility of stroke patients, and these interventions effectively improves muscle strength, motor coordination, balance, and psychological status. In addition, the exercise interventions are able to promote brain recovery by improving neuroplasticity and neurological function, which significantly improves the patients' ability to perform activities of daily living and independence.

**Conclusion:**

Exercise intervention is an effective treatment that can significantly improve the physical activity and quality of life of stroke patients during their rehabilitation. Future studies should further explore the optimal mode of exercise intervention, focus on the development of individualized treatment plans, and incorporate new technological aids to enhance the therapeutic effect.

## 1 Introduction

Stroke has been identified by the World Health Organization (WHO) as the third leading cause of death globally, accounting for ~10% of global deaths (1), and is one of the leading causes of long-term disability, with high morbidity, disability, and mortality rates (1–3). Stroke is an acute disease in which the blood supply to the brain is interrupted or a blood vessel ruptures, resulting in damage to brain tissue. Depending on the etiology, stroke can be classified as ischemic stroke (caused by the narrowing or occlusion of blood vessels supplying blood to the brain, resulting in insufficient blood supply to the brain) and haemorrhagic stroke (caused by rupture of a blood vessel in the brain, resulting in cerebral hemorrhage) (4)^1^. With advances in medical technology, the acute treatment of stroke has improved significantly, but two-thirds of stroke survivors must still cope with the neurological deficits that exist, and only 20% of patients are able to return to a normal daily life (5). The most common impairments following stroke are motor deficits, such as contralateral limb hemiparesis, which affects more than 80% of stroke patients and more than 40% of patients in the chronic phase (6). Upper limb motor deficits include muscle weakness, changes in muscle tone (spastic state), joint contractures, laxity or impaired motor control. These impairments have an impact on activities of daily living, such as reaching, picking up objects, grasping objects, or using tools such as mobile phones, which in turn limits the patient's ability to care for themselves and participate in society (7, 8).

In recent years, exercise intervention, as an effective means of rehabilitation, has become an important way to restore motor function and improve physical activity in stroke patients. More and more studies have shown that appropriate exercise intervention can not only improve the motor function of stroke patients, but also effectively improve their daily activities, quality of life, and mental health. The aim of this review is to investigate the effects of exercise intervention on physical mobility of stroke patients and to review the progress of related studies in recent years. By analyzing different types of exercise intervention strategies and their effects, it will provide theoretical support for clinical practice and suggest future research directions and potential room for improvement.

## 2 Literature search

This study was conducted by searching Google Scholar, PubMed, Web of Science, Scopus and CNKI databases from January 2000 to March 2025 to obtain relevant research results in the last 25 years. The search keywords included “stroke,” “exercise intervention,” “physical mobility,” “aerobic exercise,” “strength training,” “balance training,” “functional training,” “physical functioning,” “rehabilitation,” etc. Combined search was performed using Boolean logic operations (AND, OR). Inclusion criteria: (1) the study subjects were stroke patients; (2) the interventions were mainly related to exercise training; (3) the outcome indexes involved physical mobility or functional improvement; (4) clinical trials, observational studies, systematic reviews and meta-analyses were included. Exclusion criteria: (1) interventions unrelated to exercise; (2) study subjects who were not stroke patients; (3) unofficially published literature; and (4) literature with incomplete data or poor study design.

## 3 Impact on physical mobility after stroke

The effects of physical activity after stroke are far-reaching and multifaceted, involving physiological function, psychological state and social participation (9–11). As stroke often leads to neuronal damage in specific areas of the brain, patients usually experience limb weakness, hemiparesis or partial paralysis, and reduced motor abilities (12, 13). The resulting immobility of the limb and reduced use of the affected muscles can lead to disuse atrophy, which can be further contributed to by a lack of muscle contraction and mechanical stress. However, changes in motor control and coordination can be achieved through neuroplasticity, which refers to the ability of the brain to rewire itself (12, 14). However, in some cases, neuroplasticity may negatively affect muscle activation and coordination, leading to an imbalance in muscle loading, which in turn triggers muscle atrophy (14, 15). Haemorrhagic stroke has a range of negative effects on muscle health by triggering hemorrhage within or around the brain, directly damaging brain tissue and neural pathways (12, 16). In addition, hemorrhage induces an inflammatory response in the brain and surrounding tissues, leading to the release of cytokines and other inflammatory molecules. These inflammatory molecules not only affect nerve signaling, but also promote degradation of muscle proteins (12, 17).

In addition, stroke may also lead to the development of dystonia abnormalities, which include both spasmodic dystonia and spastic state (18–20). Spasmodic dystonia is defined as involuntary or sustained tonic contraction of the muscles in the presence of upper motor neuron lesions (19), whereas the spastic state is a velocity-dependent increase in muscle tone caused by the overexcitation of the tonic stretch reflex in patients with upper motor neuron injury (20). Both phenomena affect patients in many ways, causing interference with quality of life and function in daily activities as well as increasing the risk of joint contractures. As a result of prolonged functional limitations in physical activity, patients are prone to joint stiffness, muscle atrophy, and decreased bone density, which further limits the recovery of their physical mobility (21–23).

Sensory dysfunction and cognitive dysfunction are also key factors affecting the physical mobility of stroke patients (24, 25). Numerous studies have shown that sensory dysfunction in stroke patients is reflected in touch, pain, temperature, and proprioception (26, 27), while cognitive dysfunction is mainly reflected in thinking, language, memory, attention, perception, planning, decision-making, and problem-solving abilities (28). All of these disturbances can have a dramatic impact on the patient's physical mobility and can increase the risk of injury. Over time, they may also lead to mood disorders (e.g., anxiety, depression, etc.), which in turn may affect the patient's overall quality of life (29, 30). In addition, it has been shown that socio-environmental factors can also affect the physical mobility of stroke patients (31, 32).

In summary, the factors affecting the physical mobility of stroke patients are complex and multifaceted, including not only the impairment of physiological activity functions, but also the indirect effects of psychological, sensory, cognitive and social aspects. The interaction of multiple factors makes the functional recovery after stroke complex and long-term, highlighting the importance of comprehensive rehabilitation interventions to maximize patients' quality of life and social participation.

## 4 Effects of exercise on physical mobility in stroke patients

Stroke is an acute cerebrovascular event that usually results in the loss of function in parts of the brain, leading to a range of motor and cognitive impairments. Loss of muscle strength, impaired motor coordination, balance problems, and limitations in daily physical activities are the most common sequelae of stroke. These problems not only severely affect the patient's ability to care for themselves, but also lead to significant challenges in the recovery process. Exercise rehabilitation plays a crucial role in this process, helping patients to regain some of their lost functions and facilitating their reintegration into daily life. Therefore, this paper will explores the effects of different exercise modes on the physical mobility of stroke patients.

### 4.1 Effects of aerobic exercise on physical mobility of stroke patients

As one of the diseases with the highest disability rate in the world, stroke causes physical activity dysfunction, which brings a heavy burden to patients and their families. Although traditional rehabilitation means can improve patients' motor ability to a certain extent, they often have problems such as long period, high cost and limited functional recovery. With the development of sports medicine, aerobic training, as a safe, low-cost and repeatable intervention, has gradually received attention in the field of clinical rehabilitation.

Studies have shown that aerobic training has a positive contribution to a number of motor functions in stroke patients. A study by Pinheiro et al. (33) found that short-term aerobic cycling training significantly improved lower limb muscle strength, walking speed, balance, mobility, and functionality in patients. However, as the intervention lasted for 5 days, its effect may be influenced by the mechanisms of spontaneous recovery in the early post-stroke period (34). In addition Jin et al. (35) 24 sessions of 60-min aerobic cycling intervention training, the results further verified that the training could effectively improve the patients' muscle activation and control ability, which verified the value of aerobic exercise in stroke rehabilitation.

In addition to respiratory function, water aerobic training also showed positive effects. An intervention study showed that stroke patients' thoracic expansion capacity and exertional lung volume (MVV) were significantly improved after receiving water aerobic training for 40 min four times a week for 12 weeks (36). Considering that stroke patients are often accompanied by respiratory muscle weakness or central regulatory deficits, such improvements could help to reduce dyspnoea, prevent pulmonary complications, and possibly promote brain tissue repair and neurological reconstruction by elevating the level of oxygen supply (37, 38).

In addition to improvements in physical functioning, aerobic exercise has shown favorable psychological intervention effects. Aguiar et al. (39) conducted aerobic treadmill walking training for 12 weeks in 22 chronic stroke patients and found that aerobic treadmill walking training improved depression, endurance, and mobility, although it did not significantly change the level of physical activity and the duration of low-energy-expenditure activities in the patients. Also a study by Aidar et al. (40) noted that water aerobic training was effective in alleviating depression and anxiety symptoms in patients. Despite the limited effect of short-term or low-intensity aerobic training on the improvement of motor function and daily living ability of stroke patients, it has a positive effect in alleviating patients' depressive symptoms and enhancing sleep quality (41).

In summary, the introduction of aerobic training in the process of stroke rehabilitation can promote the recovery of body functions in a multidimensional way, covering the comprehensive improvement of motor ability, respiratory function and psychological state, thus significantly improving the overall quality of life of patients. As an evidence-based rehabilitation tool, aerobic exercise has shown promising clinical applications in strengthening muscle strength, improving balance, enhancing cardiorespiratory endurance, and alleviating mood disorders.

Overall, although the optimal intervention programme may vary among individuals, the available evidence supports that moderate-intensity aerobic training of 40 min three to four times per week for 6–12 weeks is effective in improving physical mobility and psychological wellbeing of stroke patients. Future studies should further investigate its optimal intervention intensity, frequency and long-term effects to optimize rehabilitation strategies for stroke patients.

### 4.2 Effects of strength training on physical mobility of stroke patients

Strength training, as a training method to enhance muscle strength and endurance through resistance exercises, plays a crucial role in the rehabilitation process of stroke patients. Post-stroke patients often face problems such as muscle weakness, balance disorders, and decreased physical mobility, and strength training can effectively improve these dysfunctions and promote the recovery of patients' physical mobility through targeted resistance exercises (42–44).

Strength training has been found to help improve a number of motor functions in stroke patients. Weiss et al. (45) found that progressive resistance training significantly increased lower limb strength and hip extension on both the affected and intact side of the patient, as well as improved motor performance and balance. Shao et al. (42) further validated the positive effects of strength training on non-hemiplegic measures in their study, significantly increasing the patient's strength on the hemiplegic side of the body after a 6-week intervention (43, 44). After 6 weeks of intervention, it significantly increased the patients' muscle strength in the eccentric biceps brachii, iliopsoas, and quadriceps muscles, and there was also a significant increase in balance and daily activities. This suggests that functional recovery can be indirectly facilitated by non-affected side training for the directly trained affected side.

In a separate case study, strength training was found to increase walking endurance and lower limb thrust, validating the potential role of strength training in physical recovery recovery^2^.

Kim et al. (46) and Gharib et al. (47) explored the effects of isokinetic strength training in stroke patients in separate studies and found limited improvement in walking speed, but significant gains in muscle strength, increased peak knee and ankle torque, and improvements in gait and TUG testing were clear, suggesting that isokinetic training may be more targeted to optimize muscle strength and motor performance in patients.

Furthermore, in a maximal strength training exercise, significant gains in affected vs. intact measured lower limb strength and improvements in Timed-Up-And-Go time and 6-min walk distance were found after 8 weeks of intervention, but did not change four-step squared test time, aerobic status, or quality of life in patients with chronic stroke, suggesting that maximal strength training may be beneficial for specific motor functions but has a whole-body endurance or quality-of-life limited impact, and that a more integrated training programme may be required to achieve full rehabilitation (48). Notably, strength training is not only beneficial for physical function, but may also improve depression and anxiety in stroke patients (49, 50). This has important implications for the prevention and reduction of mood disorders in post-stroke patients.

In summary, different forms of strength training have shown positive trends in enhancing muscle strength, improving motor performance and balance, and may promote neurological recovery and mood regulation through direct or indirect pathways. However, more high-quality randomized controlled trials are needed to validate their optimal intervention modalities, duration, intensity grading and long-term follow-up effects.

### 4.3 Effect of balance training on physical mobility of stroke patients

After stroke, patients often suffer from balance dysfunction as a result of central nervous system damage, which is manifested by decreased postural control, gait asymmetry, and increased risk of falling. Relevant studies have reported that balance training, which includes stretching and weight shifting, adjusting the response of exercise to changes in body movement, and strengthening lower limb muscles, is an important form of exercise for improving balance in stroke patients (51–54).

Studies have shown that balance training is effective in improving a variety of motor functions (balance function, walking efficiency, and dynamic postural control, etc.) in stroke patients. Chen et al. (55) found significant improvements in dynamic balance, self-care, and sphincter muscles after balance training interventions in their study. In a study of a balance control trainer, patients showed significant improvements in the 10-meter walk test (10 mWT), the TUG test, and the Berg Balance Scale (BBS) after 2 weeks of intervention, and these effects were even more pronounced after 4 weeks of intervention (56). Another study similarly showed that balance training significantly improved patients' BBS, TUG and functional independence (57). These studies suggest that even short-term balance training interventions can produce significant improvements in several functional indicators.

In addition, a systematic meta-analysis clearly indicated that balance training is not only safe and feasible, but also has broad applicability to balance and walking function in stroke patients, and it is recommended for stroke patients associated with decreased balance (58).

Of interest, robot-assisted balance training has demonstrated unique benefits in stroke patients. Inoue et al. (54) showed that robot-assisted balance training significantly improved Mini-BESTest scores in early stage stroke patients (1 week−3 months), yielding better results than conventional balance training with traditional rehabilitation programs. However, most current studies have focused on patients 6–12 months post-stroke and usually require some ability to stand or walk, and less on early balance training. Early intervention may have a more significant rehabilitation effect, especially for early recovering stroke patients, and robot-assisted balance training has demonstrated significant benefits (59, 60).

In summary, the available evidence suggests that balance training has favorable benefits in the rehabilitation of stroke patients, especially in terms of dynamic kinetics. However, most studies have focused on patients longer after stroke, and there is a paucity of studies on earlier interventions. Further studies are needed to verify whether balance training can lead to better rehabilitation outcomes at an earlier stage. In addition, although robot-assisted training has shown better results, more field studies are needed to assess whether it is suitable for all patients, the cost of its implementation, and its operability. Therefore, future studies could focus more on the clinical application of early balance training as well as the generalisability and feasibility of robot-assisted rehabilitation techniques to further improve stroke rehabilitation treatment protocols.

### 4.4 Effects of functional training on physical mobility of stroke patients

Functional training, as a training method based on daily activities, has gradually become an important part of stroke rehabilitation therapy. It helps to restore patients' physical mobility by simulating and reinforcing their basic movement patterns in daily life and plays a key role in improving their self-care ability (61–63).

Functional training has been found to have a positive effect in improving motor function, balance, and quality of life of stroke patients. Zhu et al. (64) found that 8 weeks of functional training was able to significantly improve walking speed, balance, and functional mobility, and Ghasemi et al. (65) also observed in their study that functional training was effective in improving muscle tone and gait performance in chronic spastic stroke patients. Some studies further noted that functional training combined with rehabilitation therapy also significantly improved upper limb motor function, joint mobility, and ability to perform activities of daily living, as well as reducing patients' anxiety and depression (66).

In their study, Hashidate et al. (67) found that functional training increased leg strength and gait indices (functional extension test, timed “get up and go” test, comfortable gait speed and maximum gait speed) over a 6-month intervention, although the effects were significantly correlated with time to stroke onset or Brunnstrom recovery stage. Although the effects were significantly correlated with stroke onset time or Brunnstrom recovery stage, overall physical function was improved. In contrast, in a robot-assisted functional training, the intervention was found to increase step length, joint extension, and balance test score aspects of the MMAS, BBS, and TUG (68).

In summary, functional training, as a rehabilitation method based on daily activities, has shown remarkable effects in the rehabilitation of stroke patients. Whether applied alone or in combination with other rehabilitation tools, it shows great potential in promoting the physical recovery of stroke patients, and its comprehensive improvement of muscle strength, balance, gait and psychological status makes it an effective intervention strategy worthy of clinical dissemination, especially when combined with technological assistive devices that may further optimize the effectiveness of rehabilitation. Future studies could further explore the optimal intensity, duration and individualization of the intervention to better guide practice and enhance its therapeutic value.

## 5 Potential mechanisms for the effect of exercise on physical activity in stroke patients

### 5.1 Neuroplasticity

Neuroplasticity is the ability of the nervous system to adapt to changes in the internal and external environments at the structural and functional levels, including the remodeling of dendrites and axons, the enhancement or weakening of synaptic connections (synaptic plasticity), and changes in the expression of relevant neurotrophic factors. Exercise, as an effective non-pharmacological intervention, can stimulate and mobilize the potential of neuroplasticity through multiple mechanisms, such as enhancing neuronal excitability, promoting synaptic transmission efficiency, and up-regulating the expression of neurotrophic factors, thus promoting the structural remodeling and functional recovery of the damaged brain areas, and providing an important support for the recovery of central nervous system diseases.

#### 5.1.1 Remodeling of dendrites and axons

Dendrites are the main structures for neurons to receive signals, and their remodeling helps to enhance the connection and information transmission between neurons, which is an important basis for neuroplasticity and functional recovery. Brain-derived neurotrophic factor (BDNF) is an important nerve growth factor widely expressed in the central nervous system, which is synthesized in the form of the glycosylated precursor protein BDNF (proBDNF) and post-translationally converted to the mature form of BDNF (mBDNF) (69). It has been shown that aerobic exercise increases the ratio of BDNF/proBDNF in the ischaemic hippocampus of rats, and that the balance between BDNF/proBDNF plays a key role in dendritic spine plasticity, which positively affects depression in stroke patients (70, 71). The number of dendritic spines in the peri-infarct cortex, hippocampus, and the expression of post-synaptic density protein (PSD-95), synaptophysin, BDNF, and TrkB were significantly increased after treadmill exercise, which improved motor function and transient memory in mice (72). In addition, the HITT 3-week-ahead intervention appeared to promote BDNF expression and delivery in brain and plasma via the PGC-1α pathway after cerebral ischaemia, which may positively affect mobility in stroke patients (73).

Axonal remodeling is an important part of neural regeneration after cerebral ischemia and is an important marker of changes in neuroplasticity. Exercise training downregulates the expression of Nogo-A, Nogo66 receptor-1 (NgR1), and Rho-A in the peri-infarct region of the cortex and promotes axonal repair in hypertensive stroke rats (74). Constraint-induced movement therapy (CIMT) may promote remodeling of motor cortex neurons, neurofilaments, dendritic/axonal regions and myelin sheaths by partially inhibiting the Nogo-A/NgR/RhoA/ROCK pathway, and increase the expression of growth-associated protein 43 (GAP-43), synaptophysin, vGlut1, and PSD-95 in the disinnervated cervical spinal cord, which may promote the improvement of motor function in hemiplegic cerebral palsy mice with improved motor function (75, 76). In addition, the expression of protein kinase C (PKC), GAP-43 and serine 41-phosphorylated GAP43 (p-GAP43) was significantly increased after exercise, which was associated with recovery from exercise-induced paralysis (77). The interaction of PKC and GAP43 has been associated with remodeling of connectivity and neuronal plasticity in cerebral infarcted rats (78).

#### 5.1.2 Changes in synaptic plasticity and expression of related neurotrophic factors

Synaptic plasticity plays a crucial role in the recovery of neurological function after stroke. After stroke, whole or specific regions of the brain become ischaemic and hypoxic, which causes neuronal and synaptic damage and leads to neurological dysfunction (79). Both mild and strenuous exercise regimens can induce synaptic plasticity and improve motor and cognitive function in stroke patients by modulating the expression of relevant proteins such as GAP-43, HIF-1α, and BDNF (80). Treadmill training induces the expression of calcium/calmodulin-dependent protein kinase II α isoform (Camk2a) through the upregulation of cytoplasmic FMR1-interacting protein 1 (CYFIP1), which enhances synaptic plasticity and dendritic remodeling of the ischemic hemi-dark band, thereby improving functional recovery in stroke mice (81). Rehabilitation increases the stability of new synapses formed in the first weeks after infarction to an extent that correlates with improvements in skilled motor performance (82). Excitatory neurotransmission and its activity, dependence, and plasticity are mainly determined by the AMPA receptor (AMPAR) (83). CIMT increases the number of functional synapses of GluR2 and the expression of AMPARs on the surface of the hippocampal post-synaptic membrane in the affected sensory-motor cortex and thus promotes the motor function of the impaired upper limb after stroke by enhancing the ischaemic hemisphere's AMPARs-mediated synaptic transmission recovery (84). NMDA receptors (NMDARs) are ligand-gated ion channels assembled from two GluN1-essential subunits and two subunits belonging to the GluN2 family (GluN2A to GluN2D), which are widely distributed in the brain, and play important roles in synaptic development, synaptic transmission, long-term plasticity, and neural network activity (85, 86). It has been shown that pre-ischemic running exercise reduces brain damage by decreasing the overexpression of PKC-α, increasing the expression levels of GLT-1, Akt, and PI3K, and decreasing the expression of mRNA for NR2B and mGluR5 (87). After HIIT training in ischemic stroke rats, increased levels of GluN2B protein as well as decreased levels of GluN2A protein in hippocampal tissues were found to improve hippocampal neuroplasticity and alleviate the depressive state in rats (88).

### 5.2 Muscle strengthening

**Figure 1:**
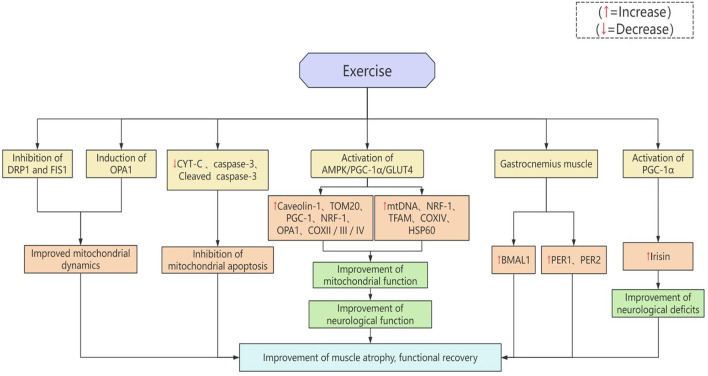
Potential mechanisms of exercise enhanced muscle function.

Stroke patients often exhibit significant motor dysfunction, which not only stems from damage to the central nervous system, but is also closely related to skeletal muscle atrophy, decreased strength and impaired neuromuscular control. Exercise intervention, as an important component of stroke rehabilitation, can promote overall motor recovery by strengthening muscle function through a variety of mechanisms in addition to promoting brain plasticity Figure 1. It was found that treadmill training could increase the expression levels of Caveolin-1, TOM20, PGC-1, NRF-1, OPA1, and COXII/III/IV by activating the AMPK/PGC-1α/GLUT4 signaling pathway, reduce cerebral oedema, improve the function of mitochondria, promote their biosynthesis, and enhance energy metabolism, which not only helped to reduce the volume of cerebral infarction and improve neurological function, but also contributed to the reduction of cerebral infarction volume and improve neurological function, but also may alleviate post-stroke muscle atrophy and enhance muscle metabolism and functional recovery at the skeletal muscle level (89–92). Voluntary exercise improves mitochondrial dynamics by inhibiting DRP1 and FIS1 expression and inducing OPA1 expression, and inhibits the mitochondrial apoptotic pathway by down-regulating the expression of CYT-C, cleaved caspase-3, and caspase-3, which ameliorates muscle atrophy (93). HIIT enhances the serum and paralyzed muscle PGC-1α pathway-related factors in rats with stroke expression and release, and its expression level in skeletal muscle is closely related to its concentration in blood. Activation of the PGC-1α pathway after cerebral ischaemia induces upregulation of irisin and correlates with improvement of neurological deficits, suggesting that it may serve as a potential marker for paralyzed muscle repair in stroke (94). In addition, the expression of PGC-1 was significantly up-regulated after 3 days of treadmill training, and the levels of mtDNA, NRF-1, TFAM, COXIV, and HSP60 were all significantly increased after training lasted for 7 days, suggesting that exercise promotes mitochondrial biosynthesis after ischemic injury, which in turn alleviates muscle atrophy and improves the behavioral performance of rats (95). The mRNA levels of BMAL1, PER1 and PER2 were up-regulated in gastrocnemius muscle on both sides after treadmill training. Among them, the expression of BMAL1 was significantly increased on the hemiplegic side, the expression of PER1 was decreased, and the expression of PER2 was significantly increased on the non-hemiplegic side, and it was found that treadmill training helped to alleviate muscle atrophy and regulate the expression of bioclamp-related genes in the skeletal muscle (96).

### 5.3 Inflammatory response

**Figure 2:**
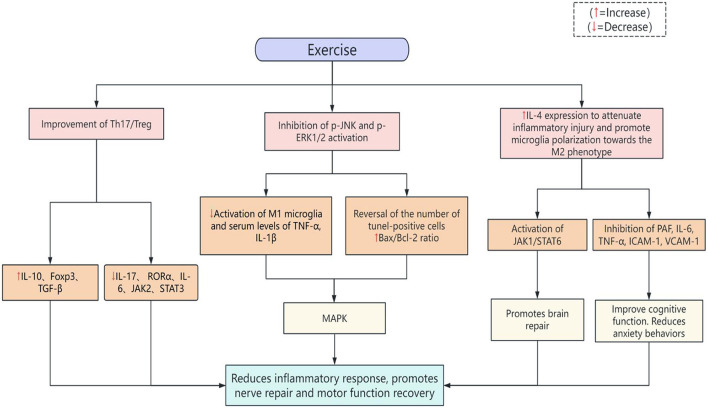
Potential mechanisms for exercise to alleviate the inflammatory response.

After stroke, the central nervous system rapidly initiates an intense immune-inflammatory response. Although this process is beneficial to damage clearance, it may also enlarge the infarct foci, inhibit neuroplasticity, and exacerbate motor dysfunction. More and more studies have shown that scientific exercise interventions can alleviate the harmful inflammatory response through multiple mechanisms, thus promoting nerve repair and motor function recovery Figure 2. Studies have shown that treadmill training can effectively improve stroke-induced Th17/Treg immune imbalance, upregulate the expression of anti-inflammatory factor IL-10 and transcription factor Foxp3, and downregulate the levels of pro-inflammatory factor IL-17 and transcription factor RORα. In terms of inflammatory response, treadmill training significantly decreased the concentrations of IL-6 and IL-17 and increased the levels of IL-10 and TGF-β, suggesting that exercise has a modulating effect on post-stroke inflammation. Further mechanistic studies showed that treadmill training inhibited the phosphorylation activity of JAK2 and STAT3, and its neuroprotective effect may improve motor function by modulating the JAK2/STAT3 signaling pathway, thereby attenuating neuronal apoptosis, inflammatory response and Th17/Treg imbalance (97). Exercise effectively reduced infarct area, neurological deficit score and brain damage in stroke rats by inhibiting the activation of p-JNK and p-ERK1/2. Further studies found that post-stroke exercise reduced the activation of M1 microglia and serum levels of TNF-α and IL-1β, reversed the increase in the number of tunel-positive cells and the Bax/Bcl-2 ratio, and attenuated the inflammatory response and apoptosis in the stroke rats by inhibiting the MAPK pathway, resulting in the improvement of function (98). Aerobic exercise intervention significantly down-regulated the expression levels of pro-inflammatory cytokine IL-1β and chemokine MCP-1 in brain tissues of post-stroke rats and improved motor function, prevented neuronal cell death and inhibited the activation of microglia and astrocytes, suggesting that exercise has a certain role in regulating inflammatory responses after cerebral ischaemia (99). Treadmill exercise could attenuate inflammatory injury by increasing IL-4 expression and promoted microglia polarization toward the M2 phenotype, facilitating brain repair, while IL-4 may activate downstream of the JAK1/STAT6 pathway (100). In addition, it was also able to significantly inhibit PAF, IL-6, TNF-α, ICAM-1, and VCAM-1, which significantly improved cognitive function, attenuated anxiety-like behaviors, and promoted post-stroke brain function and motor recovery in stroke rats (101).

### 5.4 Angiogenesis

**Figure 3:**
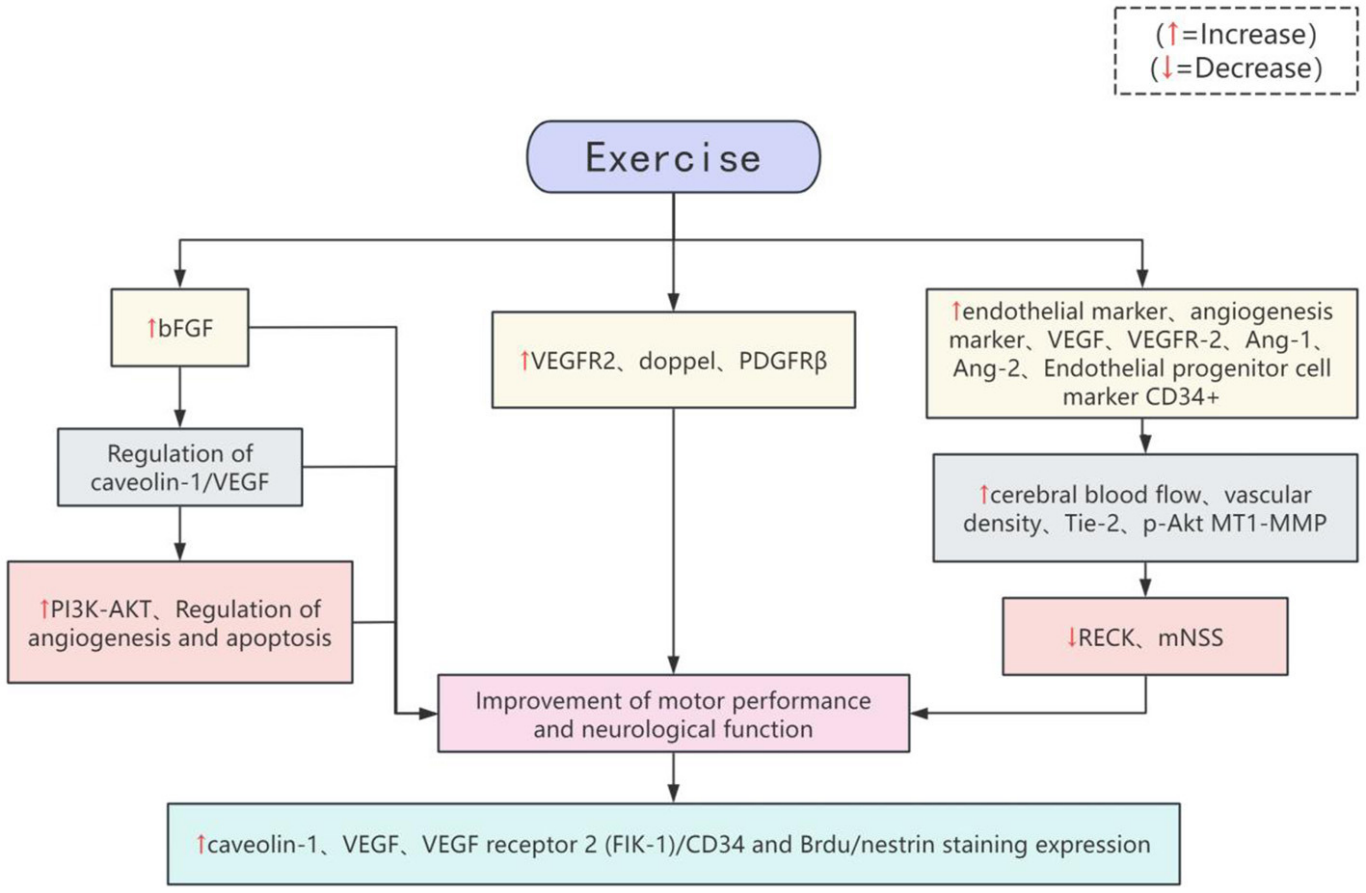
Potential mechanisms by which exercise regulates angiogenesis.

Angiogenesis plays a crucial role in the recovery of mobility after stroke, mainly through improving the blood supply of brain tissue, promoting neural repair will and enhancing neuroplasticity to help patients recover motor function Figure 3. It was found that treadmill training promoted the expression of bFGF, improved neurological recovery, reduced infarct volume, and also enhanced the expression of caveolin-1, VEGF, VEGF receptor 2 (FIK-1)/CD34, and Brdu/nestrin staining. Small interfering RNA targeting bFGF blocked the protective effect of bFGF. Furthermore, recovery 4 weeks after stroke still ameliorated ischaemia-induced injury without the need for bFGF shRNA, suggesting that treadmill training may aid functional recovery by modulating the caveolin-1/VEGF pathway in the ischaemic zone (102). Treadmill training increases the expression of angiogenesis-related proteins VEGFR2, doppel, and PDGFRβ in the peri-infarct and corresponding contralateral motor cortex, which promotes the recovery of motor function after stroke (103). Treadmill training promotes and regulates angiogenesis and apoptosis through the PI3K-AKT pathway, reduces infarct size, and improves neurological function and motor performance in rats (104). In addition, exercise training increased the levels of endothelial markers and angiogenic markers, VEGF, VEGFR-2 and Ang-1, Ang-2, and endothelial progenitor cell marker CD34^+^, and increased cerebral blood flow, vascular density, and the expression of Tie-2, p-Akt, and MT1-MMP in ischemic areas of the rats, whereas the levels of RECK expression and mNSS scores were significantly reduced. Improvement of motor performance and neurological function, reduction of infarct area as well as increase in the number of surviving cells in the peri-infarct area, and a tendency for prolongation of motor persistence in stroke rats (105–108).

## 6 Precautions

Before conducting exercise intervention for stroke patients, a comprehensive assessment of their health status should be carried out to actively prevent risks such as disease recurrence and secondary diseases, which is more conducive to the recovery of the diseases.

It is found through the literature reviews that: the most common symptom of the vascular system in patients with hyperthyroidism is atrial fibrillation (109), so the relevant indexes of thyroid function should be monitored before stroke patients undergo rehabilitation. Ion channel function should also be monitored, as abnormalities in ion channel function may lead to normo-QT syndrome, which affects atrial electrophysiology and is also closely related to the risk of atrial fibrillation (110).

Atrial fibrillation is the most common predisposing factor to the risk of stroke, and the incidence of stroke due to atrial fibrillation remains high even when treated with oral anticoagulants (111–113). Therefore, prevention of atrial fibrillation reduces the incidence of stroke to a certain extent, and it becomes more meaningful to actively intervene in the causes of stroke. With respect to lifestyle interventions, evidence indicates that the incidence of atrial fibrillation is higher among individuals who smoke and consume alcohol compared to non-smokers and non-drinkers (114–117).

Regarding physical activity, intense sports activities, whether long-term endurance training or occupation-related activities, appear to increase the risk of atrial fibrillation recurrence. The underlying mechanisms remain unclear but may be associated with changes in atrial structure, such as dilation and fibrosis (118). Research has shown that intense exercise may induce right ventricular remodeling, which is the adaptation process of the heart to function and structure under long-term endurance exercise. If atrial fibrillation patients cannot effectively control their exercise volume, it may lead to increased cardiac burden and adversely affect health (119, 120). Therefore, patients with a history of stroke and atrial fibrillation require stricter control of exercise intensity during training to prevent increased cardiac burden and reduce the risk of atrial fibrillation recurrence. Exercise for stroke patients should primarily focus on aerobic activities. However, unfortunately, there is currently no established threshold for exercise intensity and duration that can effectively prevent AF while also providing cardiovascular benefits.

## 7 Summary and outlook

### 7.1 Summary

Exercise interventions significantly improve the physical mobility of stroke patients by improving coordination, muscle strength, balance, and neurological function. The benefits of exercise are not only in terms of physiological improvements, but also in terms of cognitive and emotional benefits. Different types of exercise show different mechanisms of action in improving physical mobility in stroke patients and play a crucial role in restoring physical mobility in stroke patients, but there are still many unexplored areas that deserve further research with the aim of providing more effective and comprehensive rehabilitation strategies for stroke patients.

### 7.2 Outlook

Looking to the future, the use of exercise interventions in post-stroke rehabilitation is promising. With a deeper understanding of exercise physiology and neuroscience, future research will increasingly focus on designing personalized treatment plans that take into account the specific conditions and needs of patients, leading to more precise exercise intervention strategies. At the same time, as technology advances, tools such as virtual reality, robot-assisted rehabilitation and smart wearables are likely to become new adjuncts to exercise interventions. These technologies can improve the effectiveness of therapeutic exercise and increase patient awareness of their condition through accurate monitoring and real-time feedback. In addition, major directions for future research in stroke rehabilitation will include the combined use of exercise and other therapeutic approaches, as well as the evaluation of long-term outcomes. At the same time, there is a need to deeply explore how exercise affects the mechanisms of neuroplasticity and repair, so as to better promote the rehabilitation effect. Through interdisciplinary collaboration and technological innovation, exercise interventions are expected to play an important role in helping stroke patients restore function and improve their quality of life.

## Data Availability

The original contributions presented in the study are included in the article/supplementary material, further inquiries can be directed to the corresponding authors.

## Footnotes

^1^Unnithan AKA, Mehta P. *Hemorrhagic Stroke*. (2020).

^2^Schmidt A. *The Effect of Power Training Coupled with High-Intensity Gait Training to Improve Functional Mobility in a Patient with Right Sided Hemiparesis Following a Stroke*. (2024).
